# Green odor attenuates a cold pressor test-induced cardiovascular response in healthy adults

**DOI:** 10.1186/1751-0759-2-2

**Published:** 2008-01-15

**Authors:** Takakazu Oka, Sota Hayashida, Yuko Kaneda, Masaki Takenaga, Yoko Tamagawa, Sadatoshi Tsuji, Akikazu Hatanaka

**Affiliations:** 1 Psychosomatic Medicine Division, Department of Neurology, University of Occupational and Environmental Health, Japan. Iseigaoka 1-1, Yahatanishi-ku, Kitakyushu, 807-8555, Japan; 2 Department of Biochemistry, Yamaguchi University, Yamaguchi, 753-8511, Japan

## Abstract

**Background:**

Green odor, a mixture of equal amounts of 2*E*-hexenal (leaf aldehyde) and 3Z-hexenol (leaf alcohol) has been demonstrated to have an anti-stress effect in rats. This study investigated whether or not green odor also has an anti-stress effect in humans.

**Methods:**

Changes in blood pressure, heart rate, and the skin temperature of a fingertip were observed after presenting green odor at a concentration of 0.03% or vehicle via inhalation through the nose for 10 min to eight healthy normotensive adults. We also assessed the pleasantness of green odor and its effect on mood states via assessment with the Profile of Mood States (POMS) questionnaire. Cardiovascular response to green odor and the vehicle were compared among 11 additional healthy adults by use of the cold pressor test.

**Results:**

Of 19 subjects, 15 (79%) reported that the green odor was pleasant. Green odor had no effect on blood pressure, heart rate, skin temperature, or POMS score under non-stressful conditions. In the second experiment, green odor attenuated cold pressor test-induced increases in systolic and diastolic blood pressure and facilitated the recovery of skin temperature.

**Conclusion:**

These findings suggest that green odor has an anti-stress effect in healthy humans.

## Background

The odor of green leaves arises from eight volatile compounds that include 3Z-hexenol (leaf alcohol) and 2*E*-hexenal (leaf aldehyde) [1]. These compounds are synthesized in the chloroplast membrane in green leaves and have a wide variety of biological effects including acting as plant-to-plant messengers in allopathy, insect-attracting pheromone-like substances, and bactericidal-like substances [2] (for review, see [3]). It has been reported that the odor of a mixture containing equal amounts of 3Z-hexenol and 2*E*-hexenal, ''green odor,'' has an anti-fatigue effect in rats [4] and humans [5]. 3Z-hexenol, a component of green odor, is reported to have an anxiolytic effect in mice [6] and to decrease the amplitude of P300 activity in humans [7]. Recent studies have demonstrated that green odor has an anti-stress effect in rats. For example, green odor blocked immobilization stress-induced adrenocorticotropic hormone (ACTH) release [8] and cage change stress-induced hyperthermia [9].

We hypothesized that green odor also has an anti-stress effect in humans. In the present study, we assessed the effect of green odor on blood pressure (BP), heart rate (HR), and skin temperature (Ts), as well as subject perception of odor pleasantness and a possible change in mood under non-stressful conditions. We then assessed the effect of green odor on cardiovascular response during the stress of a cold pressor test. In this report, we demonstrate for the first time that green odor does exhibit an anti-stress effect in humans.

## Methods

### Subjects

Nineteen healthy normotensive adults without any disorder of the olfactory system participated after giving informed consent. In experiment 1, eight subjects participated (four men, four women) age 23–46 years (31.8 ± 3.1 years, mean ± standard error of the mean (SEM)). In experiment 2, a different eleven subjects participated (six men, five women) age 21–48 years (34.5 ± 3.7 years). All subjects were requested to avoid using fragrant cosmetics or having strong smelling meals on the day of the experiment.

### Methods

Experiments were carried out in a quiet, air-conditioned room kept at 23–24°C between 1 p.m. and 5 p.m. Experiments were conducted twice under both conditions with green odor and with a vehicle, with the same subjects, at the same time on different days at least seven days apart. Subjects were randomly divided into two groups to ensure counterbalance.

Experiments were approved by the Ethics Committee of the University of Occupational and Environmental Health, Japan and conducted in accordance with their guidelines.

#### Experiment 1: Effects of green odor on perception of pleasantness, mood state, and cardiovascular parameters under non-stressful conditions

After entering the experiment room, the subject was asked to sit on a chair and relax for 20 min. Meanwhile, a Japanese version of the Profile of Mood States (POMS) survey [10] was filled in. POMS is a self-rating questionnaire used for measuring six mood states: tension-anxiety (T-A), depression (D), anger-hostility (A-H), vigor (V), fatigue (F), and confusion (C). Automatic monitoring of BP and HR from the left arm was then done each minute for at least seven minutes total. Skin temperature (Ts) of the ventral surface of the right index fingertip was also monitored continuously. When BP, HR, and Ts became stable, values were recorded for three min. An average of three recordings served as the baseline value. After baseline measurement, a sheet of filter paper (30 × 150 mm) was impregnated with an aliquot of test solution containing 100 mg green odor or vehicle and was held 2 cm from the tip of the nose for presentation. Ten min after exposure of test solution, the paper was removed. BP, HR, and Ts were monitored for an additional five min. After the experiment, subjects again completed the POMS and rated the intensity and pleasantness of the odor. Odor intensity (0–5) and degree of pleasantness (-4 – +4) were assessed based on a six-point rating scale of odor intensity and a nine-point rating scale of pleasantness/unpleasantness, respectively. These scales are commonly used by the Ministry of the Environment and described elsewhere (Table 1) [7]. BP and HR were measured by a sphygmomanometer (ListMini, Nippon Colin Co. Ltd., Tokyo Japan). Ts was recorded continuously by a copper-constantan thermocouple that was connected to a digital thermometer (ThermoController, Unique Medical, Fukuoka, Japan).

**Table 1:** Six-point rating scale of odor intensity and nine-point rating scale of pleasantness/unpleasantness.

(1) Six-point rating scale of odor intensity	(2) Nine-point rating scale of pleasantness/unpleasantness.
0: Odorless	-4: Extremely unpleasant
1: Barely perceptible odor	-3: Highly unpleasant
2: Faint but source-discriminative odor	-2: Unpleasant
3: Easily perceptible odor	-1: Slightly unpleasant
4: Strong odor	O: Equivocally pleasant
5: Extremely strong odor	+1: Slightly pleasant
	+2: Pleasant
	+3: Highly pleasant
	+4: Extremely pleasant

### Preparation of green odor

Green odor, a mixture of equal amounts of 2*E*-hexenal and 3*Z*-hexenol, was diluted with the odorless solvent triethyl citrate (TEC) to a concentration of 0.03% (w/w). TEC served as the vehicle. 2*E*-hexenal, 3*Z*-hexenol, and TEC were generous gifts from Soda Aromatic Co. Ltd., Tokyo.

#### Experiment 2: Effect of green odor on cold pressor test-induced changes in BP, HR, and Ts

This experiment was performed in the same room as was used for experiment 1. After entering the experiment room, the subject sat on a chair and relaxed for at least 20 min. BP, HR, and Ts were then monitored automatically each min for at least seven min. When these values became stable, they were recorded for a further three min. An average of three values was used as the baseline value. Subsequently, subjects were presented with green odor or vehicle as was done in experiment 1. The subjects then immersed their right hand, past the level of the wrist, for one min in a bucket filled with slushy ice water (4°C) (cold pressor test, CPT). Ten min after starting the CPT, the paper was removed and BP, HR, and Ts were observed for an additional 20 min. After recording was finished, the subject rated the intensity and unpleasantness of pain experienced during CPT on a 0–10 numerical rating scale, in which 0 indicates "no pain" or "not unpleasant" and 10 represents "the worst possible pain imaginable" or "the worst unpleasantness." Subjects also rated the intensity and pleasantness of the odor.

### Statistical analysis

Values were expressed as means ± SEM. The intensity and pleasantness of the odor were examined by the Mann-Whitney U test. POMS scores and CPT-induced pain intensity and discomfort were examined by the paired *t*-test and Wilcoxon signed-rank test. Differences in BP and HR from baseline to one min following CPT (the maximum response time) were calculated and between group differences in these "deltas" were analyzed by the paired *t *test. Differences in Ts from baseline were calculated for every one min interval up to 10 min following completion of CPT, and the area under the curve between 0 min to 10 min following CPT (AUC_0–10 min_) was calculated and between group comparisons were done using the paired *t-*test.

## Results

### Effect of green odor on pleasantness and mood state

The intensity and pleasantness of the green odor and vehicle are shown in Table 2, which summarizes data obtained from experiments 1 and 2. Subjects reported green odor to be more intense (p < 0.01) and more pleasant (p < 0.05) than the vehicle, TEC. Of 19 subjects, 15 (79%) reported that green odor was slightly pleasant, pleasant, or highly pleasant. One subject reported that green odor was slightly unpleasant. However, this subject also reported that the vehicle was slightly unpleasant, so the response to green odor may not have been due to the green odor itself, but rather to TEC (Table 2). The effects of green odor on POMS scores are shown in Table 3. Neither green odor nor TEC changed POMS subscale scores.

**Table 2:** Intensity and pleasantness/unpleasantness of green odor and vehicle.

Rating of odor intensity **	0	1	2	3	4	5
Green odor (n)	0	0	5	10	4	0
Vehicle (n)	2	4	9	4	0	0

Rating of pleasantness/unpleasantness *	-1	0	1	2	3	4

Green odor (n)	1	3	5	8	2	0
Vehicle (n)	1	9	5	4	0	0

N = 19. **:p < 0.01; *:p < 0.05.

**Table 3:** The effect of green odor on mood states under non-stressful conditions.

POMS subscale	Pre-exposure	Post-exposure	p value
T-A	9.9 ± 2.3	8.9 ± 1.9	n.s.
D	10.4 ± 4.1	10.1 ± 4.4	n.s.
A-H	8.0 ± 2.1	6.9 ± 1.9	n.s.
V	10.9 ± 1.9	12.0 ± 2.5	n.s.
F	7.3 ± 1.5	7.8 ± 1.7	n.s.
C	6.9 ± 1.2	8.3 ± 1.8	n.s.

N = 8. Mean ± SEM. n.s.: not significant. POMS: Profile of Mood States; T-A: tension-anxiety; D: depression; A-H: anger-hostility; V: vigor; F: fatigue; C: confusion.

### Effect of green odor on BP, HR, and Ts under non-stressful conditions

Changes in systolic blood pressure (SBP), diastolic blood pressure (DBP), HR, and Ts were compared between exposure to the green odor and to the vehicle. In Table 4, baseline values and values at 1 min and 10 min after presentation of odor are shown for comparison with the results of experiment 2. No parameter was significantly different between green odor and vehicle at any observation point. Furthermore, SBP, DBP, HR, and Ts at 10 min did not differ from baseline values for either exposure (Table 4).

**Table 4:** The effect of green odor on physiological parameters under non-stressful conditions. Subjects were exposed to green odor or vehicle for 10 minutes.

	Green odor	Vehicle	p-value
Systolic blood pressure (mmHg)			
At baseline	105.6 ± 3.2	106.4 ± 5.0	n.s.
1 min	104.9 ± 4.8	106.0 ± 4.1	n.s.
10 min	108.4 ± 5.1	108.3 ± 4.5	n.s.

Diastolic blood pressure (mmHg)			
At baseline	59.3 ± 3.3	61.3 ± 4.3	n.s.
1 min	58.9 ± 3.5	59.6 ± 4.2	n.s.
10 min	54.0 ± 5.1	60.4 ± 4.1	n.s.

Heart rate (beats/min)			
At baseline	69.4 ± 2.7	66.4 ± 1.6	n.s.
1 min	71.0 ± 4.0	63.7 ± 2.2	n.s.
10 min	70.5 ± 2.8	64.8 ± 1.3	n.s.

Skin temperature (°C)			
At baseline	32.2 ± 2.8	32.9 ± 3.5	n.s.
1 min	33.0 ± 2.3	32.8 ± 3.6	n.s.
10 min	31.1 ± 4.3	33.2 ± 3.4	n.s.

N = 8. Mean ± SEM. N.s.:not significant.

### Effects of green odor on CPT-induced cardiovascular responses

**Figure 1:**
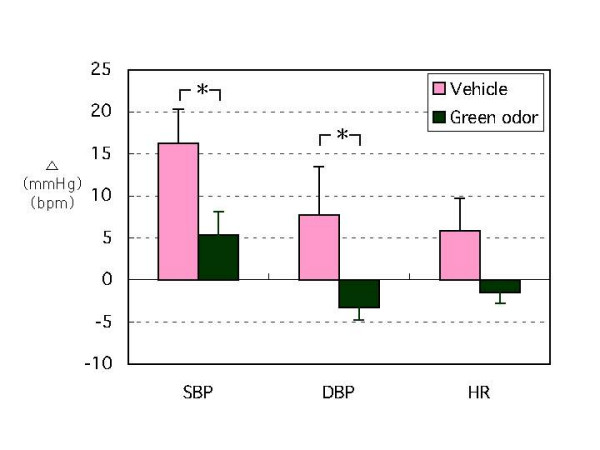
The effect of green odor or vehicle on CPT-induced changes in SBP, DBP, and HR. Changes in SBP, DBP, and HR one min after the start of CPT versus baseline levels (ΔSBP, ΔDBP, and ΔHR) were evaluated. N = 9. Means ± SEM. *: p < 0.05. Abbreviations: CPT, cold pressor testing; SBP, systolic blood pressure; DBP, diastolic blood pressure; HR, heart rate.

**Figure 2:**
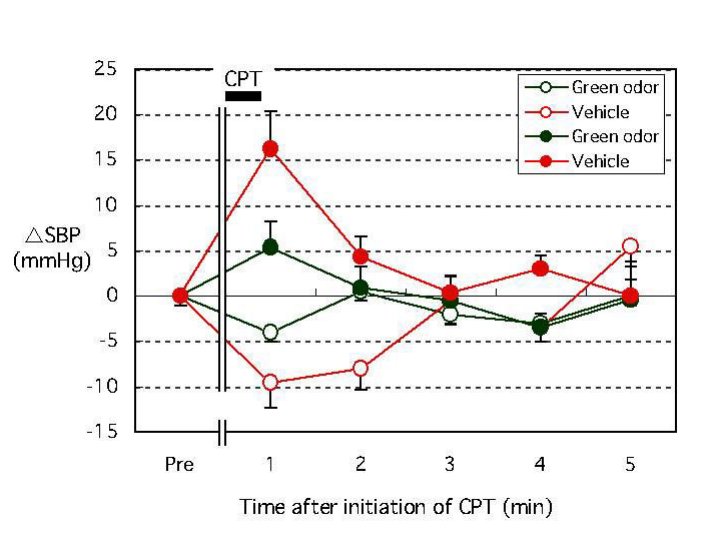
The effect of green odor or vehicle on CPT-induced changes in SBP. Changes in SBP (ΔSBP) in subjects whose SBP increased (filled circle, n = 9) after initiation of CPT and in subjects whose SBP decreased (open circle, n = 2) after initiation of CPT are shown. Means ± SEM. Abbreviations: CPT, cold pressor testing; SBP, systolic blood pressure.

Baseline values for SBP, DBP, and HR during exposure to green odor were 112.8 ± 3.5 mmHg, 62.1 ± 3.2 mmHg, 68.6 ± 3.2 bpm, and 33.5 ± 0.6°C, respectively. Values for subjects exposed to vehicle were 113.6 ± 4.3 mmHg, 61.6 ± 3.8 mmHg, 71.1 ± 3.7 bpm, and 34.1 ± 0.4°C, respectively (no difference in baseline values). Both SBP and DBP increased just after initiation of the CPT in 9 of 11 subjects. BP readings were highest one min after starting the CPT and returned to baseline levels within three min. Figure 1 compares changes in SBP, DBP, and HR at one min after initiation of CPT from baseline levels (ΔSBP, ΔDBP, ΔHR) for green odor and vehicle exposures. At one min after the start of the CPT, ΔSBP for green odor was 5.3 ± 2.8 mmHg, whereas it was 16.2 ± 4.1 mmHg for the vehicle. The ΔDBP for green odor was -3.3 ± 1.2 mmHg, whereas it was 7.7 ± 3.0 mmHg for the vehicle. Both ΔSBP and ΔDBP were significantly smaller for the green odor group than for the vehicle group (p < 0.05) (Fig. 1). In contrast, BP decreased for two subjects after initiation of CPT. Their CPT-induced decrease was most evident one min after the start of the test; this change was also attenuated with green odor exposure. CPT-induced changes in the SBP of all subjects are shown in Figure 2.

**Figure 3:**
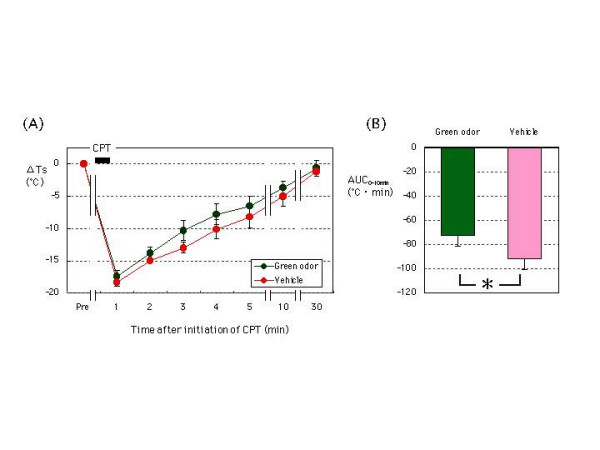
The effect of green odor or vehicle on recovery of baseline Ts following initiation of CPT. (A) Changes in Ts after the start of CPT from baseline level (ΔTs) were compared for green odor and vehicle. (B) The AUC_0–10 min _was compared for green odor and vehicle. N = 11. Means ± SEM. *: p < 0.05. Abbreviations: Ts, skin temperature; CPT, cold pressor testing.

Ts decreased one min after initiation of the CPT and returned gradually to baseline level within 30 min for all subjects under both exposures (green odor and vehicle). Changes in Ts from the baseline value (ΔTs) were compared for green odor and vehicle. The ΔTs one min after initiation of CPT were -18.0 ± 0.8°C for green odor and -18.9 ± 0.6°C for vehicle. The ΔTs one min after initiation of CPT did not differ between exposures. Figure 3 shows the calculation and comparison of AUC_0–10 min _values for the two exposures. The AUC_0–10 min _value for green odor (-72.9 ± 8.6°C·min) was significantly different from that for vehicle (-92.0 ± 9.3°C·min) (p < 0.05), suggesting that green odor facilitates recovery of Ts to the baseline level during the CPT condition.

### Effect of green odor on pain intensity and unpleasantness

**Figure 4:**
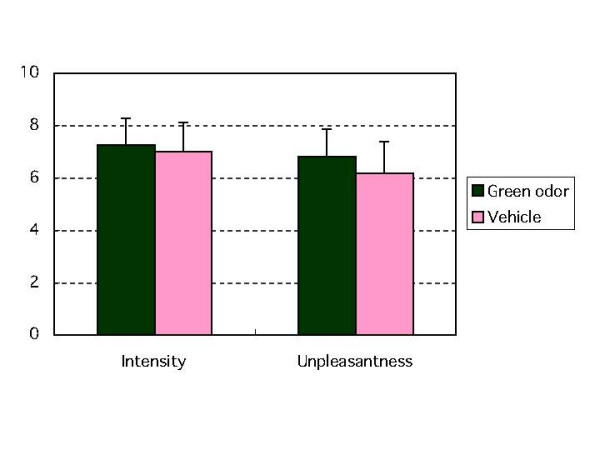
The effect of green odor or vehicle on intensity and unpleasantness of pain induced by cold pressor testing.

Most subjects perceived the CPT to be painful. When exposed to green odor, subjects rated CPT-induced pain intensity to be 7.3 ± 1.0 compared with 7.0 ± 1.1 when exposed to vehicle. They rated unpleasantness of pain as 6.8 ± 1.0 when exposed to green odor compared with 6.2 ± 1.2 when exposed to vehicle. Neither pain intensity nor the unpleasantness of pain differed for exposure to green odor versus vehicle (Fig. 4).

## Discussion

The present study demonstrated that green odor attenuated the increase in SBP and DBP induced by CPT and facilitated recovery of Ts without having any effect on these parameters, under non-stressful conditions. These findings are the first to suggest that green odor exhibits an anti-stress effect in healthy, normotensive human adults.

The inhibitory effect of green odor on cardiovascular response to the CPT may not be a secondary effect due to its hypoalgesic action [4] because the scores for CPT-induced pain intensity and unpleasantness did not differ for green odor versus vehicle. A previous study reported that green odor increased the mechanical pain threshold in humans [4]. It is unknown why the effect of green odor on CPT-induced pain obtained in the current study is different from the findings obtained in the previous study. One possible explanation is that the other research group assessed the effect of green odor on mechanical pain threshold, whereas our study investigated the effect on thermal pain. The effect of green odor on pain might differ depending on modality. The anti-stress effect associated with green odor does not appear to be induced secondarily through change in an emotional state such as reduced anxiety or anger, because neither the tension-anxiety nor hostility-anger scores of the POMS changed with exposure to green odor.

Stress is characterized by activation of the hypothalamic-pituitary-adrenocortical (HPA) axis and sympathetic-adrenomedullary system (SAM). In rats, green odor has been found to attenuate immobilization stress-induced increases in plasma ACTH levels [8] and reduce psychological stress-induced hyperthermia [9]. As the sympathetic nervous system plays an important role in psychological stress-induced hyperthermia [11-13], findings from these animal studies suggest that green odor has an inhibitory effect on stress-induced activation of both the HPA axis and the SAM. In this study, green odor attenuated the CPT-induced increase in BP.

In hypertensive patients, the sympathetic nervous system is over-reactive, and stress-induced increases in BP are greater than in healthy subjects. Therefore, in some hypertensive patients, psychological stress makes blood pressure difficult to control. This characteristic of green odor, to attenuate a thermal stress-induced change in BP, might be beneficial in treating hypertensive patients whose BP readings increase dramatically under conditions of psychological stress [14]. In contrast, interestingly, BP decreased following initiation of CPT in two subjects. It is uncertain why these subjects showed a different stress response pattern from the other subjects. However, green odor attenuated the CPT-induced decrease in BP in these subjects. Thus, it appears that green odor may inhibit stress-induced decreases in BP as well as increases in BP.

### Concentration of green odor

In the present study, we investigated the effect of green odor at a concentration of 0.03% for the following reasons. Firstly, animal studies have demonstrated that green odor at this concentration has an anti-stress effect [8,9]. Secondly, our pilot study, conducted before the currently reported work, showed that the concentration range at which the majority of subjects reported green odor as pleasant was around 0.03%. In that study, we examined the pleasantness of equal amounts of 3Z-hexenol and 2*E*-hexenal at different concentrations: 0.003%, 0.03%, and 0.3%. Twenty percent of the subjects reported 3Z-hexenol and 2*E*-hexenal at 0.3% to be extremely strong and unpleasant (data not shown). Even though 0.3% green odor may have a stronger anti-stress effect than at a lower concentration, the odor may not be clinically applicable as stress-reducing aromatherapy if subjects find it unpleasant. Therefore, we decided to conduct our major experiments using a concentration that would probably not be uncomfortable for most subjects: Indeed, the 0.03% concentration of 3Z-hexenol and 2*E*-hexenal was perceived as pleasant by 79% of subjects. However, as the next step in our research, it will be important to investigate the dose-dependency of green odor's anti-stress effect to determine the concentration that exhibits maximal effect and to elucidate if green odor still has an anti-stress effect in adults who find green odor unpleasant.

### Possible neural mechanisms

The precise mechanisms in the central nervous system through which green odor exerts its anti-stress effect are not fully understood. Forced swimming stress-induced expression of Fos-immunoreactivity in the paraventricular nucleus of the thalamus (PVT) of rats was attenuated by green odor [15]. The PVT is thought to play a pivotal role in modulating forebrain processing of stress-related information and is also implicated directly or indirectly in the generation of behavioral, endocrine, and autonomic responses to stress [16,17]. Therefore, it is possible that the anti-stress effect of green odor is achieved through inhibition of PVT neurons [15]. A positron emission tomography study using rhesus monkeys demonstrated that green odor activated the anterior cingulate gyrus as well as olfactory-related regions such as the prepyriform area, substantia innominata, and orbitofrontal cortex [18]. Given the crucial role of the anterior cingulate gyrus in processing emotion and affective behavior [19], activation of its neurons may also contribute to the anti-stress effect of green odor.

## Conclusion

This study suggests that green odor has an anti-stress effect in healthy adults and may have potential for clinical aromatherapy.

## Abbreviations

ACTH: Adrenocorticotropic hormone; BP: Blood pressure; HR: Heart rate; Ts: Skin temperature; POMS: Profile of Mood States; T-A: Tension-anxiety; D: Depression; A-H: Anger-hostility; V: Vigor; F: Fatigue; C: Confusion; TEC: Triethyl citrate; CPT: Cold pressor test; AUC: Area under the curve; SBP: Systolic blood pressure; DBP: Diastolic blood pressure; HPA: Hypothalamic-pituitary-adrenocortical; SAM: Sympathetic-adrenomedullary system; PVT: Paraventricular nucleus of the thalamus.

## Competing interests

The author(s) declare that they have no competing interests.

## Authors' contributions

TO designed the study protocol and drafted the manuscript. TO, SH, YK, MT, and YT conducted the experiments and analyzed the data. ST supervised the experiments. AH supervised the whole green odor project. All authors read and approved the final manuscript.
